# Is Loop Ileostomy in Patients with Cecal Bascule a Viable Option?

**DOI:** 10.1155/2019/8549692

**Published:** 2019-07-22

**Authors:** Tushar Shetty, Tommy Ivanics, Hassan Nasser, Amalia Stefanou

**Affiliations:** ^1^Wayne State University School of Medicine, Detroit, MI, USA; ^2^Department of Surgery, Henry Ford Hospital, Detroit MI, USA; ^3^Division of Colon and Rectal Surgery, Department of Surgery, Henry Ford Hospital, Detroit, MI, USA

## Abstract

**Background:**

Cecal bascule, initially described in 1899 by Treves, is the rarest form of cecal volvulus and represents a phenomenon when a redundant and distended cecum folds anteriorly over the ascending colon causing an intestinal obstruction. Patients with cerebral palsy are at increased risk for this condition.

**Case Presentation:**

We present a 28-year-old male with cerebral palsy, functionally dependent in all activities of daily living, who had undergone a loop ileostomy for cecal bascule. He then presented to our emergency department with a large loop ileostomy prolapse, which was the result of an inverted prolapsed cecum through the efferent ileostomy limb. He underwent a right hemicolectomy with end ileostomy and transverse mucous fistula creation through the previous ostomy site. He progressed well appropriately postoperatively and was discharged home.

**Conclusions:**

While cecal bascule is a rare form of bowel obstruction, patients with cerebral palsy are at an increased risk for this condition. The treatment options are numerous and are primarily surgical. Diverting loop ileostomy alone is not a recommended treatment. A high index of suspicion is warranted in all cases of large bowel obstruction to minimize risk of recurrence, morbidity, and mortality for patients afflicted by this condition.

## 1. Background

**Figure 1:**
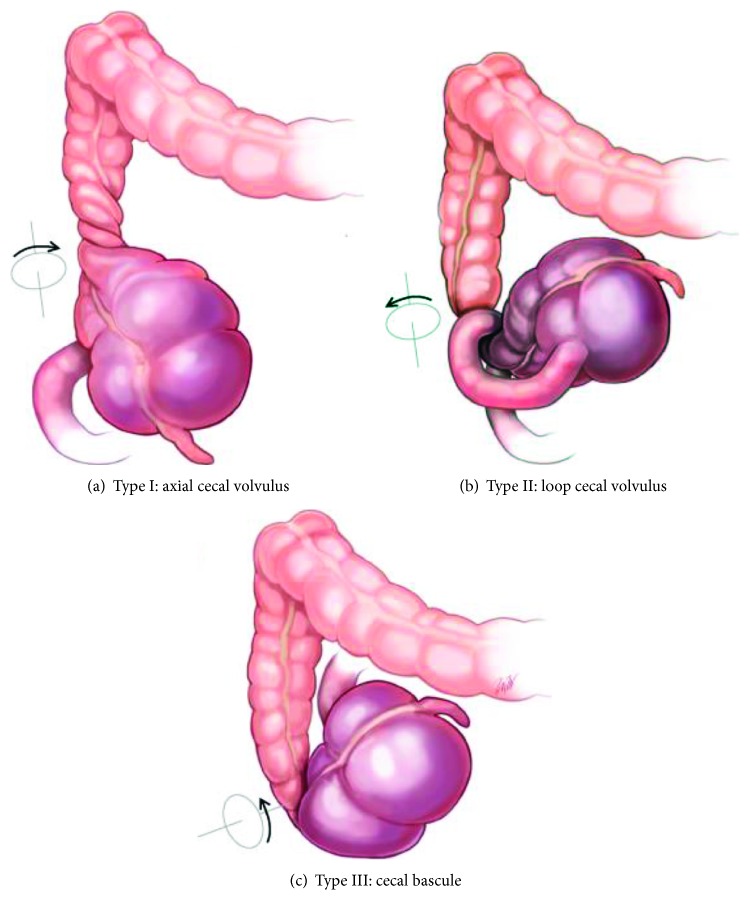
Types of cecal volvulus [2].

Cecal bascule is the rarest form of cecal volvulus, which in itself is rare accounting for 1-2% of large bowel obstructions [1–3]. While it was initially described in 1899 by Treves, Weinstein was the first to describe the radiologic and clinical findings of the condition [4]. Three types of cecal volvuli exist (Figure 1): axial, loop, and cecal bascule. The two former account for eighty percent of cases and the latter 2-20% [5]. The incidence of cecal bascule is highest in males between 35 and 75 years of age [5–7]. The French word “bascule” means rocker or seesaw and is descriptive of the pathophysiology, which relates to a large distended cecum intermittently folding anteriorly over the ascending colon [5]. The etiology may be related to an intestinal malrotation or a fixation abnormality resulting in nonfixation of the cecum and right colon to the peritoneum, which results in a mobile cecum prone to volvulus with subsequent obstruction, distension, and ischemia [8–10].

## 2. Case Presentation

A 28-year-old male presents with a past medical history significant for cerebral palsy, fully dependent in activities of daily living. He has had a lifelong history of intermittent abdominal distension and constipation. He had previously presented to an outside hospital due to low-grade fever with intractable nausea and vomiting. A computed tomography (CT) abdomen pelvis with contrast demonstrated findings consistent with pseudoobstruction vs. ileus, possibly due to cecal bascule or volvulus. Due to failure to improve with nonoperative measures, he underwent a decompressive colonoscopy to reduce the colonic distension. He was subsequently taken to the operating room where a cecal bascule was identified, per outside operative record, as well as a severely dilated small bowel and redundant colon. He underwent a diverting loop ileostomy, gastrostomy tube placement, and appendectomy. Reasons for this operative decision-making are not made known to the authors. His postoperative course was complicated by delayed return of bowel function requiring total parenteral nutrition.

**Figure 2:**
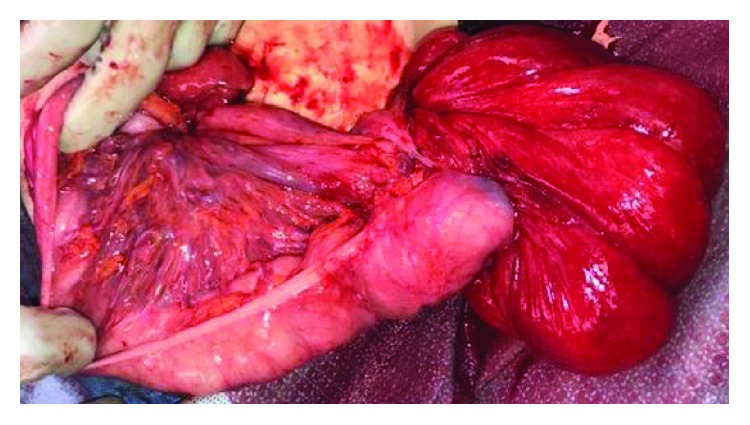
Prolapsed portion of the proximal ascending colon inverted (dark red) with extended portions of the colon (pink).

**Figure 3:**
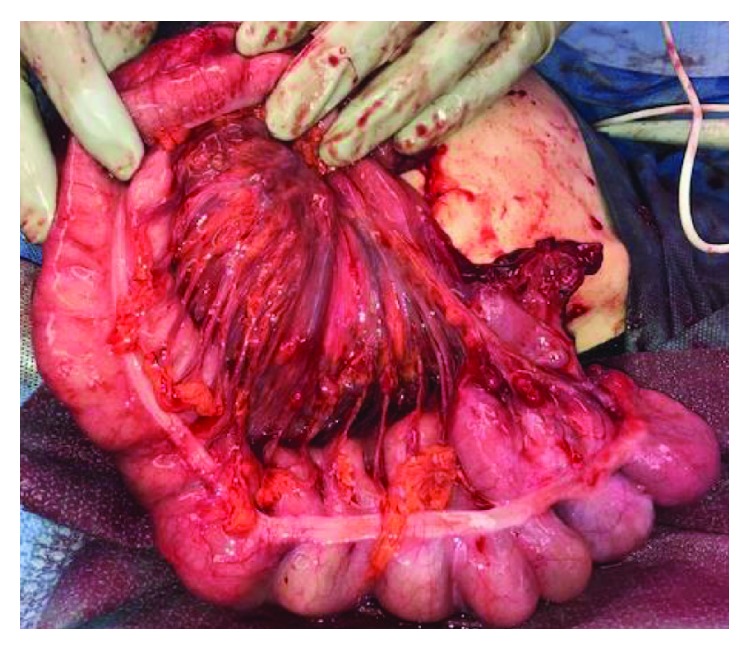
Prolapsed portion of the colon after everting the intussusception.

**Figure 4:**
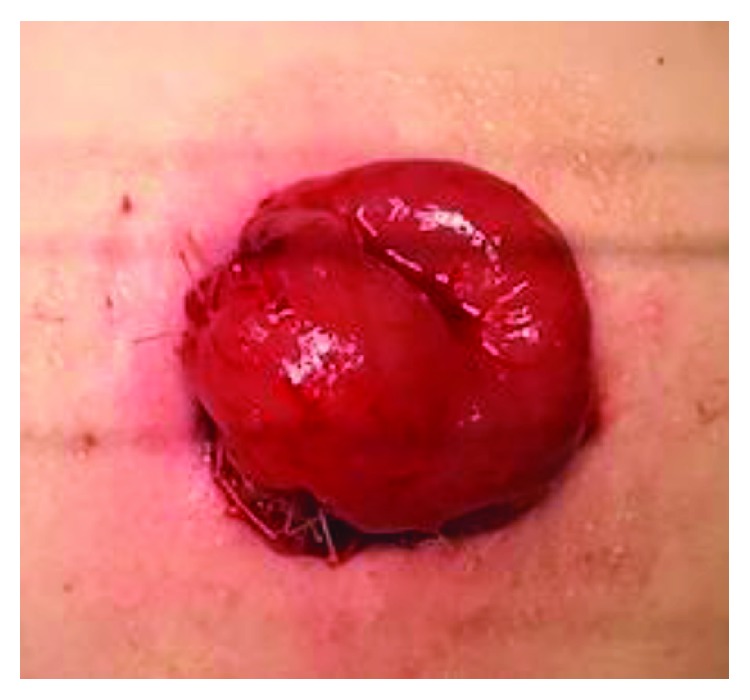
Completed ileostomy.

Approximately 6 weeks later, he presented to our emergency department with fever, leukocytosis, abdominal discomfort, and multiple episodes of emesis. He remained hemodynamically stable, but his stoma had prolapsed at least 25-30 cm and appeared edematous and dark red distally (Figure 2). He was taken to the operating room where further examination of the stoma demonstrated approximately 40 cm of prolapse. The mucocutaneous junction of the prolapsed portion was dissected to delineate anatomy (Figure 3). The mucosa was noted to be edematous and abnormal. At this point, it became apparent that the prolapsed portion was the efferent limb of the loop ileostomy with the intussuscepted cecum and the entire right colon. The decision was made to proceed with a right hemicolectomy and mucous fistula creation at the level of the transverse colon to eliminate the mobile bowel (Figures 3 and 4). This entire resection was done through the stoma site. Primary anastomosis was not performed due to the patient's poor nutritional status and need for stimulation to have a bowel movement due to spasticity. An end ileostomy and transverse colonic mucous fistula were created through the previous stoma site. The patient's postoperative course was complicated by delayed return of bowel function. He was eventually discharged home with his family on postoperative day 9 tolerating tube feeding through his gastrostomy tube with adequate ileostomy function. He was seen in the clinic postoperatively and recovered to his baseline.

## 3. Discussion

While cecal volvulus is well identified on imaging studies, cecal bascule may be more difficult to detect, particularly in patients with chronic recurrent issues with distension and constipation. This case represents a patient with what is most likely cecal bascule given his history and mobile cecum identified in the operating room. This case is unique because the entire right colon had prolapsed through the ileostomy, most likely due to increased abdominal pressure due to cerebral palsy and mobile cecum.

Patients with neurologic disease, such as cerebral palsy, and neurogenic bowel dysfunction, such as colonic pseudoobstruction (Ogilvie syndrome), are at increased risk for developing this condition likely due to bearing down and spasticity [11]. Patients present with nausea, vomiting, abdominal distension, and abdominal pain which can be either diffuse or localized to the right side of the abdomen [2, 5]. Diagnosis can be made with plain abdominal radiographs, contrast enema, or CT scans [2, 9]. The pathognomonic finding of cecal volvulus on plain film is a coffee bean-shaped air-distended loop of bowel in the left upper quadrant [2]. Delabrousse et al. from France highlighted the accuracy in CT in distinguishing between the different types of cecal volvulus. Axial torsion is characterized by a clockwise whirl sign, whereas a loop type typically includes a counterclockwise whirl sign. In contrast, CT findings characteristic of a cecal bascule is a cecum located in the central abdomen without presence of a whirl sign. The authors noted the utility of CT imaging in predicting complication from these as presence of circumferential wall thickening, pneumatosis intestinalis, increased mesenteric fat density, and pneumoperitoneum [2].

A number of treatment options exist, choices that depend on the presence or absence of bowel compromise, as well as the hemodynamic stability of the patient. While nonoperative management (through detorsion) has been described, the mainstay of therapy is typically surgery due to the high rate of recurrence [5]. A pragmatic approach to cecal volvulus is to perform an ileocecectomy or formal right hemicolectomy in patients who present with necrotic bowel. If the patient is stable, an anastomosis should be attempted. If the patient is hemodynamically unstable or has either poor nutritional or functional status at the time of resection, an end ostomy can be performed. In patients who have viable bowel and are good surgical candidates, a resection (ileocecectomy or right hemicolectomy) followed by a primary anastomosis can be performed to minimize recurrence. In an unfit patient who presents with cecal volvulus with viable bowel, a cecostomy can be attempted. However, this option is seldom performed due to the risk of recurrent volvulus [8, 12]. Loop ileostomy is not an adequate treatment.

There is little data on recurrence rates for patients who are not managed with surgical resection. For patients with cecal volvulus managed with colonoscopic reduction, the recurrence rates are upwards to 50% and therefore not recommended [13]. Patients with cerebral palsy represent an important demographic for this condition due to presence of risk factors including chronic constipation, immobility, and neurogenic bowel dysfunction [11]. Late recognition of symptoms may also contribute to morbidity and mortality, and this underscores the importance of prompt diagnosis and treatment.

## 4. Conclusions

Cecal bascule is a rare form of cecal volvulus, which itself is a rare cause of large bowel obstruction. Patients with cerebral palsy are at increased risk for this condition. The treatment options are numerous and primarily surgical with the focus on resection of the mobile bowel. Diverting loop ileostomy alone is not a recommended treatment. A high index of suspicion is warranted in all cases of large bowel obstruction and particularly in patients with neurologic diseases, to minimize risk of recurrence, morbidity, and mortality for these patients.
